# Durability of Antibody Responses to SARS-CoV-2 Vaccination over 12 Months in Pediatric Inflammatory Bowel Disease

**DOI:** 10.3390/vaccines13060549

**Published:** 2025-05-22

**Authors:** Sally J. Lawrence, Marina Viñeta Paramo, Frederic Reicherz, Jeffrey N. Bone, Zahra Jama Hussein Shire, Loujain Bilal, Gabriella Guerra, Liam Golding, Pascal M. Lavoie, Kevan Jacobson

**Affiliations:** 1 Department of Pediatrics, Division of Gastroenterology, Hepatology and Nutrition, Faculty of Medicine, British Columbia Children’s Hospital, University of British Columbia, Vancouver, BC V6H 3V4, Canada; sally.lawrence@cw.bc.ca (S.J.L.); zahra.shire@gmail.com (Z.J.H.S.); loujainbilal@gmail.com (L.B.); gabriellakguerra@gmail.com (G.G.); 2 British Columbia Children Hospital Research Institute, Vancouver, BC V5Z 4H4, Canada; marina.vineta@bcchr.ca (M.V.P.); fsreicherz@gmail.com (F.R.); jbone@bcchr.ca (J.N.B.); plavoie@bcchr.ca (P.M.L.); 3 Department of Pediatrics, Division of Neonatology, Faculty of Medicine, British Columbia Children’s Hospital, University of British Columbia, Vancouver, BC V6H 3V4, Canada; 4 Department of Pediatrics, Children’s Hospital Datteln, University of Witten/Herdecke, 45711 Datteln, Germany; 5 Department of Cellular and Physiological Sciences, University of British Columbia, Vancouver, BC V6T 1Z3, Canada; liam.golding@bcchr.ca

**Keywords:** COVID-19, vaccine, antibody, SARS-CoV-2, inflammatory bowel disease

## Abstract

Background/Objectives: Severe acute respiratory syndrome (SARS-CoV-2) has had a profound global impact and continues to represent a health challenge worldwide. The durability of SARS-CoV-2 vaccine responses in pediatric inflammatory bowel disease (PIBD) patients receiving biologic therapies is unknown. This study aimed to quantify SARS-CoV-2 antibody responses post vaccination in these immunosuppressed patients over 12 months. Methods: Prospective study comparing antibody responses against SARS-CoV-2 spike protein at 1, 3, 6, and 12 months in PIBD patients aged 5–18 years treated with anti-tumor necrosis factor alpha (anti-TNF) therapies with or without an immunomodulator (IM) versus vedolizumab. Results: Between 1 May 2021 and 1 May 2022, 194 participants on anti-TNF monotherapy (n = 78), anti-TNF with IM (n = 83), vedolizumab (n = 15), and steroids (n = 18) were recruited. Anti-SARS-CoV-2 spike levels increased after the first vaccine and were further boosted 1 month after the second dose. Linear mixed-effects modelling showed antibody waning over time (effect difference −2509 IgG AU/mL per week [95%CI: −4998–−20, *p* = 0.048]), counterbalanced by booster doses (effect difference 184,138 IgG AU/mL per additional vaccine dose [95%CI: 138,342–229,934, *p* < 0.001]). Receiving anti-TNF therapy contributed to reduced antibody responses compared to vedolizumab (anti-TNF monotherapy effect difference: −212,640 [95%CI: −336,928–−88,351] *p* = 0.001; anti-TNF with IM: −151,880 [95%CI: −277,309–−26,451] *p* = 0.018). Seroconversion and breakthrough infection rates were similar between groups, and all infections were mild, without hospitalizations. Conclusions: Although SARS-CoV-2 antibody responses were attenuated in PIBD patients receiving anti-TNF therapy compared with vedolizumab, this did not impact protection, as seroconversion and breakthrough infection rates were similar, with no hospitalizations. These data reinforce the importance of updating vaccines and, in particular, SARS-CoV-2 vaccines in immunosuppressed PIBD patients on advanced therapies.

## 1. Introduction

Severe acute respiratory syndrome coronavirus-2 (SARS-CoV-2), which causes coronavirus disease 2019 (COVID-19), has had a profound impact on society and caused the first truly global pandemic of the 21st century [1]. Over 7 million deaths globally have been attributed to COVID-19, and SARS-CoV-2 continues to exert an international burden on health systems worldwide [1,2]. In healthy children, vaccination against SARS-CoV-2 is safe and diminishes severe infection risk, hospitalization, and death associated with COVID-19 [3]. Inflammatory bowel disease (IBD) is an immune-mediated chronic inflammatory disorder that is increasing in incidence and prevalence globally in the pediatric population [4]. Children with IBD have altered underlying immune responses that may leave them vulnerable to infections [5]. In addition, although immunosuppression is the cornerstone of IBD therapy, there are concerns that this can attenuate the protective immune responses induced by vaccines [6]. Anti-tumor necrosis factor alpha (anti-TNF) and anti-integrin therapies are two of the established treatments for pediatric IBD (PIBD) [7,8]. Studies in PIBD patients treated with anti-TNF therapy have reported reduced immune responses to inactivated influenza and viral hepatitis vaccination [6]. Conversely, vedolizumab, a gut-selective anti-integrin alpha-4-beta-7 monoclonal antibody, is not associated with increased susceptibility to systemic infections or attenuated serologic responses to other pediatric vaccinations [9].

The efficacy of SARS-CoV-2 vaccination in IBD has been explored in adult studies, showing an attenuated SARS-CoV-2 vaccine response for patients treated with anti-TNF alone or in combination with an immunomodulator (IM) compared with those treated with vedolizumab [10,11,12]. We reported the short-term responses to two ancestral-strain SARS-CoV-2 doses in PIBD patients aged 12–18 years, showing an attenuated response after one dose while on anti-TNF in combination with an IM. However, after two vaccine doses, the majority of PIBD patients achieved a robust antibody response [13].

The magnitude and durability of immune responses to vaccination against SARS-CoV-2 and its variants in immunosuppressed children with IBD is currently unknown, which raises concern, especially as children under 12 years received a reduced vaccine dose. Moreover, as SARS-CoV-2 continues to evolve, it is important that we understand the role of booster vaccines in our immunosuppressed patients. The objective of this study was to evaluate the durability of antibody responses to SARS-CoV-2 messenger RNA (mRNA)-based vaccines up to 12 months after the first dose in PIBD patients treated with maintenance anti-TNF therapies alone or in combination with an IM compared to vedolizumab.

## 2. Materials and Methods

### 2.1. Participants and Recruitment

This was a prospective observational cohort study in PIBD patients followed at the British Columbia Children’s Hospital (Vancouver, BC, Canada). Study recruitment took place between 1 May 2021 and 1 May 2022. Patients aged 5–18 years were included if they had an established diagnosis of Crohn’s disease (CD), ulcerative colitis (UC), or inflammatory bowel disease unclassified (IBDU) using standard definitions of IBD, they received at least 1 dose of a SARS-CoV-2 mRNA vaccine, either BNT162b2 (Comirnaty, Pfizer-BioNTech, Pfizer—New York, NY, USA and BioNtech—Mainz, Germany) or mRNA-1273 (Spikevax, Moderna, Cambridge, MA, USA), and were treated with anti-TNF therapy or vedolizumab for at least 12 weeks at the time of first vaccination. The patients included were treated with maintenance vedolizumab or anti-TNF therapy alone or in combination with an IM (azathioprine or methotrexate). Patients treated with steroids with or without other immunosuppression were separately analyzed to compare with our patients on other immunosuppressive therapies. Participants were enrolled before receiving the first dose of vaccine and followed for 12 months. In British Columbia, the BNT162b2 vaccine dose was 30 micrograms (mcg) for children ≥ 12 years of age and 10 mcg for children 5–11 years old. The mRNA-1273 vaccine dose for the primary series was 100 mcg for children ≥ 12 years old and 50 mcg for children 6–11 years of age. The booster dose for mRNA-1273 was 50 mcg for children ≥ 12 years old, while the booster dose was 25 mcg for children 6–11 years old.

### 2.2. Serum Sample Collection

Blood collections were performed at baseline (pre-first dose) and at 1, 3, 6, and 12 months after the first dose. Serum was collected by venipuncture in gold serum separator tubes (BD Biosciences, catalog #367989), left at room temperature for clotting, and then centrifuged at 1400× *g* for 10 min and frozen at −80 °C within 4 h.

### 2.3. Serology Testing

Testing for anti-SARS-CoV-2 spike and S1 receptor-binding domain (RBD) immunoglobulin G (IgG) levels (V-PLEX SARS-CoV-2 IgG, Meso Scale Diagnostics, Rockville, MD, USA, K15575U) was performed for the ancestral strain in the whole cohort. Anti-SARS-CoV-2 spike IgG levels (V-PLEX SARS-CoV-2 IgG, Meso Scale Diagnostics, K15642U) for the Omicron subvariant (BA.5) were also measured. The Meso Scale Discovery assay threshold for antibody positivity used to define seroconversion was 1960 AU/mL for spike and 538 AU/mL for RBD, as specified by the manufacturer. The sensitivity and specificity of the assay > 15 days post vaccine was 98.3% and 99.5%, respectively. Diagnostic serology for SARS-CoV-2 infection was performed using an FDA-approved anti-SARS-CoV-2 nucleocapsid immunoassay (Elecsys^®^ Anti-SARS-CoV-2 assay, Roche, Indianapolis, IN, USA), which provided quantitative results in the form of a value based on a cutoff index (COI) or qualitative, as reactive, or non-reactive, as specified by the manufacturer.

### 2.4. Patient Variables

Variables documented included demographics (age, gender), IBD phenotype according to Paris classification, disease activity (Pediatric Crohn’s Disease Activity Index or Pediatric Ulcerative Colitis Activity Index), age at IBD diagnosis, duration and type of immunosuppression medication, SARS-CoV-2 vaccine status (type, dose, and vaccine dates), SARS-CoV-2 infection based on positive nucleic acid amplification (NAAT) or rapid antigen testing, emergency room visits, and hospitalization for SARS-CoV-2 infection post vaccination.

### 2.5. Outcomes

The primary outcome was to assess ancestral anti-SARS-CoV-2 spike IgG response at 3 and 12 months post first vaccine, comparing PIBD patients receiving maintenance vedolizumab, anti-TNF monotherapy, and anti-TNF in combination with an IM (azathioprine or methotrexate). Secondary outcomes included evaluating contributors to the SARS-CoV-2 vaccine response over time, IgG response to Omicron subvariant BA.5 at 3 and 12 months post vaccination, SARS-CoV-2 seroconversion rates at 3 and 12 months post vaccination, breakthrough SARS-CoV-2 infection after 2 vaccinations (based on positive serology or respiratory testing results [NAAT or rapid antigen testing]), hospitalization due to clinical breakthrough infection, and self-reported vaccine side effects.

### 2.6. Statistical Analysis

Discrete data were reported as numbers and percentages, and continuous data as median and interquartile range (IQR). Antibody levels between groups were log-transformed prior to analysis, and thereby, the analysis compared geometric means (gmean) with geometric standard deviation (gSD). Differences are expressed as a geometric mean ratio, representing the relative difference between groups. As this study consists of a longitudinal cohort with repeated measures for each participant at different timepoints, a linear mixed-effects regression (LMER) model was used to determine the contribution of independent predictors to the SARS-CoV-2 vaccine immunogenicity (assessed as log 10-transformed antibody concentrations). Age at first vaccine dose, type of maintenance immunotherapy, time of sample collection after first vaccine dose, number of additional vaccine doses received at time of sample collection, and SARS-CoV-2 infection status were included as fixed effects, with a random intercept for the participant. The results from this model are summarized as effect differences and corresponding 95% confidence intervals. We subsequently plotted this model, allowing for nonlinearity in trends after first vaccination by fitting the time of sample collection with a regression spline.

Patients receiving steroids were not included in the primary analysis due to the heterogeneity of the group. However, the geometric mean antibody concentration was compared between those treated with steroids (with or without other immunosuppressants) and those receiving other therapies to assess the steroid contribution to vaccine responses over time.

Pair-wise comparisons were statistically evaluated with unpaired 2-sided t-tests adjusted for multiple testing using the Bonferroni method for continuous variables and with the Chi-square test method for breakthrough infection rates. The data were analyzed with R software version 4.3.2 and were overseen by a biomedical statistician.

## 3. Results

### 3.1. Patient Characteristics

In total, 194 participants were recruited (median age 14 years [IQR 12–16], 38% female), of which 83 participants were treated with anti-TNF in combination with an IM (79% methotrexate, 21% azathioprine), 78 with anti-TNF monotherapy, and 15 with vedolizumab. Eighteen patients were treated with steroids with or without immunosuppressants. Baseline demographics and clinical characteristics of the cohorts are shown in Table 1. There were no notable demographic differences between the cohorts except that there were more UC patients in the vedolizumab group. Overall, 3% of the cohort had developed SARS-CoV-2 infection prior to any vaccinations, with similar rates between therapy groups (Table 1). There was an equal distribution of patients who had received additional vaccine doses in the first year in each group. At least two vaccine doses had been received by 95% (186/194) of participants, 65% (126/194) had received three vaccine doses, and 26% (51/194) had received four doses by the end of the study period. Of the participants, 20% (38/194) were 5–11 years old and had received a lower vaccine dose.

### 3.2. Anti-SARS-CoV-2 Spike Antibody Concentrations

**Figure 1:**
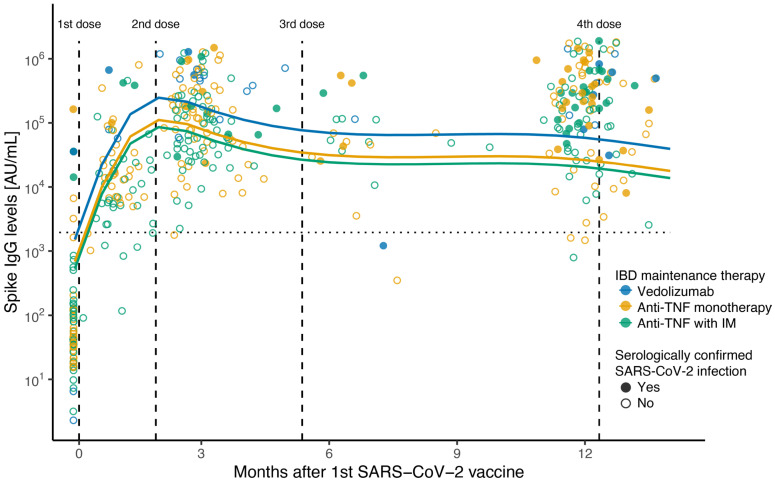
Scatterplot of anti-SARS-CoV-2 spike ancestral IgG levels after first dose of SARS-CoV-2 vaccine. Scatterplot of anti-SARS-CoV-2 spike IgG concentration (AU/mL) on a log10 scale, with fitted values from linear mixed-effects regression including a spline term for months after first vaccination. Therapy group at time of first SARS-CoV-2 vaccine: blue for vedolizumab; green for anti-TNF combined with immunomodulator; yellow for anti-TNF monotherapy. Solid circles show serologically confirmed SARS-CoV-2 infection samples; open circles show negative serology samples. Vertical lines indicate median time in days in which 2nd, 3rd, and 4th vaccines were received after 1st dose (57, 157, and 370 days, respectively). Horizontal line indicates seroconversion cut-off value (1960 AU/mL) for the Meso Scale Discovery assay.

Serological analysis post vaccination showed that anti-SARS-CoV-2 spike IgG levels increased after the first SARS-CoV-2 vaccine dose and were further boosted 1 month after the second dose. (Figure 1, Table 2). These responses were sustained over time, with a minor antibody decay (effect difference of −2509 IgG AU/mL per week [95%CI: −4998–−20, *p* = 0.048]) that was counterbalanced by the administration of booster vaccine doses (effect difference of 184,138 IgG AU/mL per additional vaccine dose [95%CI: 138,342–229,934, *p* < 0.001]) (Figure 1, Table 2). Natural SARS-CoV-2 infection was also independently associated with an enhanced anti-SARS-CoV-2 spike antibody response over time (effect difference of 151,125 IgG AU/mL [95%CI: 60,737–241,512, *p* = 0.001]; Table 2).

Although all children were able to mount an immune response after vaccination regardless of their IBD maintenance immunosuppression, spike antibody levels were significantly lower 3 months after the first vaccine dose in all therapy groups compared to the vedolizumab group in unadjusted comparisons (anti-TNF with IM IgG level mean ratio: 0.17 [95%CI: 0.10–0,30], *p* < 0.001; anti-TNF monotherapy IgG level mean ratio: 0.26 [95%CI: 0.14–1.00] *p* = 0.007); steroid therapy IgG level mean ratio: 0.08 [95%CI: 0.01–0.53] *p* = 0.013) (Table 3). However, there were no significant differences in spike antibody levels between any of the therapy groups at 12 months after the first dose compared to vedolizumab patients in unadjusted comparisons (anti-TNF with IM IgG level mean ratio: 0.43 [95%CI: 0.18–1.05]; anti-TNF monotherapy IgG level mean ratio: 0.49 [95%CI: 0.19–1.25]); steroid therapy IgG level mean ratio: 0.29 [95%CI: 0.08–1.00]; all *p* > 0.05) (Figure 1, Table 3). Still, these attenuated responses in patients receiving anti-TNF therapy were statistically significant in the LMER when adjusting for other variables (effect difference of −212,640 [95%CI: −336,928–−88,351], *p* = 0.001, and −151,880 [95%CI: −277,309–−26,451], *p* = 0.018, respectively, for anti-TNF monotherapy and anti-TNF combined with IM compared to vedolizumab) (Table 2).

In keeping with the lower vaccine dose, children < 12 years showed lower antibody levels than those 12–18 years old, which was significant at 12 months (at 3 months IgG level mean ratio: 0.66 [95%CI: 0.33–1.36], *p* = 0.647, and at 12 months IgG level mean ratio 0.27 [95%CI: 0.13–0.58], *p* = 0.004 unadjusted) (Supplementary Table S1). However, when adjusting for other LMER predictors, the age effect was non-significant (effect difference −87,383 [95%CI: −176,017–1251], *p* = 0.715) (Table 2).

Eighteen patients received systemic steroids at the time of vaccination. Spike antibody levels were numerically lower at 3 and 12 months in patients treated with steroids compared to those not on steroids (at 3 months IgG level mean ratio 0.32 [95%CI: 0.05–2.11], *p* = 0.408, and at 12 months IgG level mean ratio 0.59 [95%CI: 0.21–1.70], *p* = 0.253) (Table 3). All patients on steroids had seroconverted by 3 months and remained seroconverted at 12 months. Overall, 20% (3/15) of patients on steroids had breakthrough infections after two vaccine doses, and there were no hospitalizations.

### 3.3. SARS-CoV-2 Variant BA.5 Antibody Response

**Figure 2:**
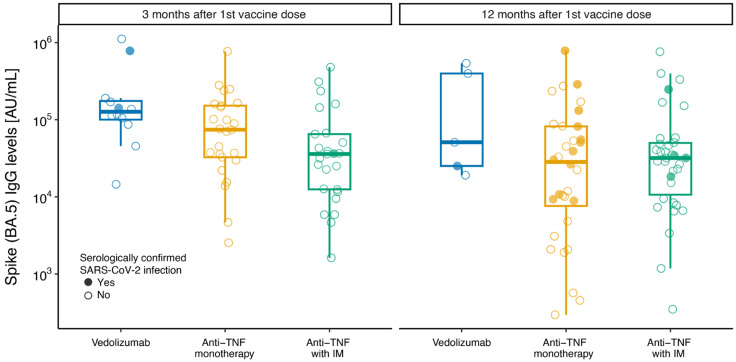
Box plots of anti-SARS-CoV-2 spike BA.5 IgG levels at 3 and 12 months after first dose of SARS-CoV-2 vaccine. Box plots of anti-SARS-CoV-2 spike (BA.5) antibody concentration (AU/mL) by therapy group according to IBD medication at time of first SARS-CoV-2 vaccine administration: “anti-TNF with IM” for anti-TNF (infliximab or adalimumab) combined with immunomodulator (azathioprine or methotrexate); “anti-TNF monotherapy” for anti-TNF (infliximab or adalimumab) monotherapy; “vedolizumab” for vedolizumab monotherapy or combination. The central line indicates median values, the boxes show the inter-quartile range (IQR), and the whiskers include the full range of values. Solid circles show serologically confirmed SARS-CoV-2 infection samples; open circles show serology-negative samples.

There was a lower spike antibody response at 3 months against SARS-CoV-2 Omicron subvariant BA.5 in patients treated with anti-TNF with IM compared with patients treated with vedolizumab (at 3 months IgG level mean ratio: 0.23 [95%CI: 0.10–0.56], *p* = 0.032, and at 12 months IgG level mean ratio 0.32 [95%CI: 0.05–2.12], *p* = 0.202) (Figure 2). Although numerically lower, there was no significant difference in antibody levels between patients on anti-TNF monotherapy compared with patients treated with vedolizumab (at 3 months IgG level mean ratio: 0.44 [95%CI: 0.19–1.04], *p* = 0.102, and at 12 months IgG level mean ratio: 0.23 [95%CI: 0.03–1.50], *p* = 0.297) (Figure 2, Table 3).

### 3.4. Seroconversion Rates Following SARS-CoV-2 Vaccination

All therapy groups reached high seroconversion rates for ancestral spike IgG levels after vaccination. At 3 and 12 months, 98.2 and 96.5% of patients on anti-TNF monotherapy had seroconverted, 100 and 98.4% of patients on anti-TNF combination therapy had seroconverted, while 87.5 and 91.7% of patients on vedolizumab had seroconverted (Supplementary Table S2).

### 3.5. Breakthrough SARS-CoV-2 Infection

Overall, 39% (73/186) of patients had evidence of a breakthrough infection after the second vaccine dose. Notably, there was no difference in breakthrough SARS-CoV-2 infections after two vaccine doses between groups (37% (30/81) of anti-TNF combination therapy, 49% (37/75) of anti-TNF monotherapy, and 40% (6/15) of vedolizumab, *p* = 0.345). No patient with infection had an emergency room visit, required hospitalization, had a serious adverse event, or died. There were no serious side effects or hospitalizations post vaccination.

## 4. Discussion

This is the largest dedicated prospective PIBD study to date investigating the long-term durability of antibody responses and protection from SARS-CoV-2 mRNA vaccination over 12 months. Our study has strengths, including a well-defined prospective cohort and the largest dedicated pediatric study detailing longitudinal antibody response and breakthrough infections after SARS-CoV-2 vaccination.

Our data show a substantial increase in antibody levels over time post vaccination for PIBD immunosuppressed patients. Our results have been corroborated in large-scale adult IBD studies that reported increasing antibody levels over time after two vaccine doses, with further benefit from additional doses [12,14,15,16,17,18,19]. Similar to previous adult studies where seroconversion rates after two doses ranged from 81 to 100%, the majority of pediatric patients seroconverted, generating durable protective antibody responses over 12 months regardless of the immunosuppression class [11,16,20,21].

Although all children were able to mount an immune response after vaccination regardless of their IBD immunosuppression, patients treated with anti-TNF therapy with or without an IM had an attenuated anti-SARS-CoV-2 spike antibody response relative to those treated with vedolizumab on multivariable modelling. Neutralization of TNF-alpha is known to disrupt T-cell-dependent humoral responses due to reduction in B-cell maturation, with a consequent reduction in antibody response [22]. IBD studies measuring antibody responses following SARS-CoV-2 vaccination have corroborated our findings in patients with anti-TNF therapy after two or three vaccine doses [10,11,12,17,18,19,23,24,25,26]. In the CLARITY IBD study, following two vaccine doses, geometric mean spike IgG antibody levels were significantly lower in infliximab-treated patients compared with those on vedolizumab. Moreover, a concomitant immunomodulator further attenuated the antibody response [24]. We also report that patients on systemic steroids had lower antibody levels, which was seen in previous studies, along with those on tofacitinib [27]. These data highlight the importance of optimizing vaccination to augment immune protection against COVID-19 in patients on anti-TNF therapy.

We identified several predictors of serological response to vaccination. Importantly, repeat SARS-CoV-2 vaccine doses were independently associated with significantly higher antibody levels, as has been reported previously [28]. In addition, SARS-CoV-2 infection promoted increased antibody levels, which mirror previous adult IBD studies [19,24,27,29]. Reassuringly, our study suggested a better SARS-CoV-2 spike IgG response to booster doses than to SARS-CoV-2 infection, which was also documented in a small PIBD study [30]. Conversely, increasing time after a vaccine dose was associated with an attenuated antibody response in keeping with the expected antibody decay. Previous studies have also reported substantial antibody decay over time [30,31]. These data support the need for repeated vaccination in immunosuppressed PIBD patients.

Importantly, age was not a factor associated with antibody response in our pediatric population despite younger patients receiving a reduced vaccine dose. Similarly, a robust response was seen in 5–11-year-old patients in another short-term study [32]. Notably, age over 60 years has been associated with diminished antibody responses, in keeping with the acknowledged effect of immunosenescence on humoral immunity [19,27].

Breakthrough infections were asymptomatic or mild, and we saw no hospitalizations during the period that corresponded to the Omicron and largest SARS-CoV-2 infection wave in British Columbia, which corroborates previous study findings [15,33,34]. This likely reflects the effectiveness of vaccine protection and the possible lower virulence of new variants. Risk factors for hospitalization in previous studies included advanced age and active IBD. In our cohort, the majority of patients were in clinical remission, which may have influenced the results [29,35]. Some adult studies have reported that patients treated with anti-TNF therapy are at greater risk of breakthrough infection than patients treated with vedolizumab [15,16,18,24]. However, the CLARITY study showed that SARS-CoV-2 antibody concentrations did not predict risk of breakthrough infection, especially with the Omicron variant [15]. Indeed, our data demonstrate that breakthrough infection rates were similar across immunosuppressant classes occurring in two fifths of all patients. Three other cohort studies saw no difference in breakthrough infection rates between immunosuppressants following two vaccine doses, including a small pediatric cohort that reported a similar breakthrough infection rate of 40% during the Omicron outbreak [26,36,37]. Notably, we used more stringent measures to assess breakthrough infection (anti-nucleocapsid antibody, PCR positivity) than most previous cohorts, which may account for the higher rates of breakthrough infections we saw. As anti-nucleocapsid antibodies may wane rapidly after an asymptomatic or mild SARS-CoV-2 infection, the true infection rate could be even higher. Yet, it is reassuring that none of these infections (either captured or not by our definition) led to hospitalization because of SARS-CoV-2 in this cohort. Moreover, no patients had severe adverse events to vaccination that required hospitalization, which correlates with data reporting that SARS-CoV-2 vaccination was safe and well tolerated, with a low IBD flare rate post vaccination [21,26,29,32,36,38,39].

Omicron and its subvariants, including BA.5, can overcome vaccine-elicited immune defenses from the ancestral vaccine strain and have increased transmissibility; therefore, investigating relative immune responses to Omicron in immunosuppressed IBD patients is important [40]. Similar to adult data, we report lower SARS-CoV-2 Omicron BA.5 antibody levels at 3 and 12 months in children treated with anti-TNF in combination with an IM versus vedolizumab [25,41]. These data highlight the importance of re-vaccination after 12 months to provide ongoing protection against variants in immunosuppressed children [42]. No difference was observed in antibody levels between patients treated with anti-TNF monotherapy and those taking vedolizumab, although the results should be interpreted with caution due to the limited sample size. It should be acknowledged that our study predated the emergence of Omicron subvariants such as XBB.1.5; however, this highlights the importance of ongoing monitoring of SARS-CoV-2 vaccine immunogenicity, as new variants continue to emerge over time.

We acknowledge several study limitations. Firstly, there was no healthy pediatric control group; however, previous data indicate that patients treated with vedolizumab have equivalent SARS-CoV-2 vaccine-induced antibody responses to healthy controls [19]. Secondly, the sample size in steroid recipients was modest, with significant heterogeneity, limiting our conclusions to a descriptive nature. This requires further prospective evaluation. Thirdly, the small samples size in the vedolizumab cohort limits generalizability in this subgroup. Finally, we looked at humoral but not cell-mediated immunity in this study, which may have underrepresented the full protective immune response; however, analysis of the effect of COVID-19 vaccines on antigen-specific T-cell responses is underway in our cohort.

## 5. Conclusions

Our study provides important data on the durability of the SARS-CoV-2 antibody response over 12 months after vaccination in children with IBD. Anti-TNF treatment attenuated serological responses to ancestral strains and the Omicron subvariant BA.5 compared to vedolizumab. Reassuringly, most pediatric patients generated a robust, durable antibody response to SARS-CoV-2 vaccines, regardless of the type of immunosuppression, with high seroconversion rates. Moreover, breakthrough infections were asymptomatic or mild, with no hospitalizations, irrespective of immunosuppression type. Importantly, additional vaccine doses augmented antibody responses counterbalancing antibody decay. Primary prevention strategies such as vaccine optimization are a crucial component in the care of IBD patients. Our study supports WHO recommendations and highlights the importance of ensuring complete vaccine schedules with appropriate vaccine updates in PIBD patients on immunosuppression, with prioritization of anti-TNF-treated patients [43]. As anti-TNF medications are being utilized to treat many immune-mediated conditions, the implications of our findings are likely relevant for a large proportion of patients with immune-mediated inflammatory conditions on advanced therapies. Moreover, these data can be used to inform vaccine decisions in pediatric patients with IBD.

## Figures and Tables

**Table 1:** Baseline characteristics of all study participants. Groups were defined according to inflammatory bowel disease (IBD) medication at time of first severe acute respiratory syndrome coronavirus-2 (SARS-CoV-2) vaccine administration.

	Full Cohort (n = 194)	Anti-TNF IM ^1^ (n = 83)	Anti-TNF ^2^ (n = 78)	Vedolizumab ^3^ (n = 15)	Steroids ^4^ (n = 18)
Age, median years (IQR) ^5^	14 (12–16)	14 (12–16)	15 (12–16)	14 (12–16)	13 (11–15)
Female, n (%)	74 (38.1)	28 (33.7)	35 (44.8)	5 (33.3)	6 (33.3)
IBD subtype, n (%)					
Crohn’s disease	135 (69.6)	66 (79.5)	53 (67.9)	5 (33.3)	11 (61.1)
Ulcerative colitis	56 (28.9)	16 (19.3)	25 (32.1)	9 (60.0)	6 (33.3)
IBDU	3 (1.5)	1 (1.2)	0 (0.0)	1 (6.7)	1 (5.6)
Disease status, n (%) ^6^					
Remission	165 (85.0)	74 (89.2)	65 (83.3)	15 (100.0)	11 (61.1)
Mild	26 (13.4)	8 (9.6)	13 (16.7)	0 (0.0)	5 (27.8)
Moderate	3 (1.5)	1 (1.2)	0 (0.0)	0 (0.0)	2 (11.1)
Severe	0 (0.0)	0 (0.0)	0 (0.0)	0 (0.0)	0 (0.0)
Anti-TNF therapy, n (%)					
Infliximab	165 (85.0)	82 (98.8)	72 (92.3)	-	11 (61.1)
Adalimumab	10 (5.0)	1 (1.2)	6 (7.7)	-	3 (16.7)
Immunomodulator, n (%)					
Azathioprine	20 (10.3)	17 (20.5)	-	1 (6.7)	2 (11.1)
Methotrexate	72 (37.1)	66 (79.5)	-	5 (33.3)	1 (5.6)
Vaccine, n (%) ^7^					
BNT162b2	174 (89.7)	76 (91.6)	67 (85.9)	14 (93.3)	17 (94.4)
mRNA-1273	3 (1.5)	3 (3.6)	0 (0.0)	0 (0.0)	0 (0.0)
Combination	17 (8.8)	4 (4.8)	11 (14.1)	1 (6.7)	1 (5.6)
Vaccine doses received, n (%)					
One	8 (4.1)	2 (2.4)	3 (3.8)	0 (0.0)	3 (16.7)
Two	60 (30.9)	24 (28.9)	25 (32.1)	6 (40.0)	5 (27.8)
Three	75 (38.7)	32 (38.6)	30 (38.5)	7 (46.7)	6 (33.3)
Four	51 (26.3)	25 (30.1)	20 (25.6)	2 (13.3)	4 (22.2)
SARS-CoV-2 infection prior to any vaccines, n (%)	6 (3.1)	2 (2.4)	3 (3.8)	1 (6.7)	0 (0.0)
Vaccine timing interval, median days (IQR)					
Between doses 1 and 2	57 (50–69)	57 (50–67)	57 (52–69)	56 (50–68)	55 (23–102)
Between doses 2 and 3	109 (82–128)	109 (82–128)	100 (82–135)	116 (84–217)	109 (43–185)
Between doses 3 and 4	204 (195–220)	205 (194–225)	210 (199–228)	189 (173–205)	215 (201–223)

^1^ Anti-TNF IM: anti-tumor necrosis factor alpha (anti-TNF) therapy combined with immunomodulator (IM); ^2^ anti-TNF: anti-TNF monotherapy; ^3^ vedolizumab: vedolizumab in mono or combination therapy with IM; ^4^ steroids: prednisone in monotherapy or in combination with any other medication; ^5^ IQR: interquartile range; ^6^ disease status defined by Pediatric Ulcerative Colitis Activity Index for ulcerative colitis and IBD unclassified (IBDU) and Pediatric Crohn’s Disease Activity Index for Crohn’s disease; ^7^ BNT162b2: Comirnaty, Pfizer-BioNTech; mRNA-1273: Spikevax, Moderna.

**Table 2:** Contributors to SARS-CoV-2 vaccine response over time. Linear mixed-effects regression model for the association between anti-SARS-CoV-2 spike IgG levels and predictor variables.

Variable	Mean Difference in Vaccine Response (95% CI)	*p*-Value
Weeks after 1st vaccine dose (per week) ^8^	−2509 (−4998–−20)	0.048
Age group ^9^		
12–18 years old	Reference	-
5–11 years old	−87,383 (−176,017–1251)	0.053
Medication		
Vedolizumab	Reference	-
Anti-TNF-IM ^10^	−212,640 (−336,928–−88,351)	0.001
Anti-TNF ^11^	−151,880 (−277,309–−26,451)	0.018
SARS-CoV-2 vaccine doses received (per dose) ^12^	184,138 (138,342–229,934)	<0.001
SARS-CoV-2 infection		
Not diagnosed	Reference	-
Self-reported	43,911 (−73,863–161,684)	0.464
Serologically confirmed	151,125 (60,737–241,512)	0.001

^8^ Weeks after 1st dose included as a continuous variable; ^9^ age group entered as a categorical variable, With age cut-off of 12 defined according to the difference in vaccine dosage between these two groups; ^10^ anti-TNF-IM; anti-tumor necrosis factor alpha therapy with immunomodulator; ^11^ anti-TNF; anti-tumor necrosis factor alpha monotherapy; ^12^ vaccine doses were included as a continuous variable.

**Table 3:** Anti-SARS-CoV-2 spike ancestral variant, RBD S1 ancestral variant, and spike BA.5 variant IgG levels 3 and 12 months after the first dose of SARS-CoV-2 vaccine. Data were compared to the vedolizumab group as the reference group, with a two-sided unpaired *t*-test on log-transformed data and Bonferroni’s correction for multiple comparisons.

		Anti-TNF-IM ^13^ (N = 63,64) ^14^	Anti-TNF ^15^ (N = 60,57)	Vedolizumab ^16^ (N = 14,11)	Steroids ^17^ (N = 8,12)
Anti-spike IgG levels (AU/mL) ^18^	3 months ^19^	79,211 (3.7) ** ^20^	120,215 (4.5) **	460,923 (2.3)	36,588 (9.4) * ^21^
	Mean ratio ^22^ (95% CI)	0.17 (0.10–0.30)	0.26 (0.14–1.00)	Reference	0.08 (0.01–0.53)
	12 months ^23^	127,094 (4.9)	144,258 (6.7)	295,595 (3.4)	85,310 (5.0)
	Mean ratio ^22^ (95% CI)	0.43 (0.18–1.05)	0.49 (0.19–1.25)	Reference	0.29 (0.08–1.00)
Anti-RBD S1 IgG levels (AU/mL)	3 months	41,743 (3.0) **	66,342 (4.1)	165,308 (2.4)	15,392 (10.8) *
	Mean ratio (95% CI)	0.55 (0.43–0.7)	0.67 (0.52–0.88)	Reference	0.36 (0.15–0.85)
	12 months	58,634 (4.0)	71,511 (4.7)	139,055 (3.5)	44,190 (3.8)
	Mean ratio (95% CI)	0.68 (0.47–1.01)	0.75 (0.51–1.12)	Reference	0.61 (0.38–0.99)
Anti-spike BA.5 IgG levels ^24^ (AU/mL)	3 months	31,600 (4.0)	60,339 (3.7)	137,062 (3.1)	NA
	Mean ratio (95% CI)	0.23 (0.10–0.56) *	0.44 (0.19–1.04)	Reference	-
	12 months	27,941 (4.8)	19,812 (7.2)	87,886 (4.8)	NA
	Mean ratio (95% CI)	0.32 (0.05–2.12)	0.23 (0.03–1.50)	Reference	-

^13^ Anti-TNF-IM: anti-tumor necrosis factor (anti-TNF) with immunomodulator; ^14^ N = X,X indicates total samples available for each timepoint (3 months and 12 months); ^15^ anti-TNF: anti-TNF monotherapy; ^16^ vedolizumab: vedolizumab monotherapy or combination therapy with immunomodulator (IM); ^17^ steroids: prednisone monotherapy or combination therapy with immunosuppressant; ^18^ antibody level geometric mean and geometric standard deviation (AU/mL); ^19^ mean 90 days after receiving the first SARS-CoV-2 vaccine; ^20^ ** *p* < 0.001 (compared to vedolizumab group) calculated on log-transformed data with 2-sided unpaired *t*-test with Bonferroni’s correction for multiple comparisons; ^21^ * *p* < 0.05 (compared to vedolizumab group) calculated on log-transformed data with 2-sided unpaired *t*-test with Bonferroni’s correction for multiple comparisons; ^22^ the mean ratio refers to the relative difference in geometric means between groups, calculated as the difference in mean on log-transformed data; ^23^ mean 360 days after receiving the first SARS-CoV-2 vaccine; ^24^ based on available samples for each therapy group (anti-TNF-IM: N = 25,34; anti-TNF: N = 25,32; vedolizumab: N = 12,5).

## Data Availability

The data underlying this article will be shared on reasonable request to the corresponding author.

## Supplementary Materials

The following supporting information can be downloaded at: https://www.mdpi.com/article/10.3390/vaccines13060549/s1, Table S1: Anti-SARS-CoV-2 spike and RBD ancestral IgG levels 3 and 12 months after the first dose of SARS-CoV-2 vaccine by age groups. Table S2: Seroconversion rates for anti-SARS-CoV-2 spike and RBD IgG ancestral levels at 3 and 12 months after first dose of SARS-CoV-2 vaccine.

## Abbreviations

Severe acute respiratory syndrome coronavirus-2: SARS-CoV-2; inflammatory bowel disease: IBD; pediatric inflammatory bowel disease: PIBD; anti-tumor necrosis factor alpha: anti-TNF: immunomodulator: IM; coronavirus disease 2019: COVID-19; messenger RNA: mRNA; Crohn’s disease: CD; ulcerative colitis: UC; inflammatory bowel disease unclassified: IBDU; BNT162b2: Comirnaty, Pfizer-BioNTech; mRNA-1273: Spikevax, Moderna; receptor-binding domain: RBD; immunoglobulin G: IgG; nucleic acid amplification test: NAAT; interquartile range: IQR; geometric mean: gmean; geometric standard deviation: gSD; linear mixed-effects regression: LMER.

## References

[1] Bennett C.M., Vally H. (2024). The evolving epidemiology of SARS-CoV-2. Microbiol. Aust..

[2] Summa K.C., Hanauer S.B. (2023). COVID-19 and Inflammatory Bowel Disease. Gastroenterol. Clin. N. Am..

[3] Olson S.M., Newhams M.M., Halasa N.B., Price A.M., Boom J.A., Sahni L.C., Pannaraj P.S., Irby K., Walker T.C., Schwartz S.P. (2022). Effectiveness of BNT162b2 Vaccine against Critical COVID-19 in Adolescents. N. Engl. J. Med..

[4] Kuenzig M.E., Fung S.G., Marderfeld L., Mak J.W.Y., Kaplan G.G., Ng S.C., Wilson D.C., Cameron F., Henderson P., Kotze P.G. (2022). Twenty-first Century Trends in the Global Epidemiology of Pediatric-Onset Inflammatory Bowel Disease: Systematic Review. Gastroenterology.

[5] Wellens J., Colombel J.F., Satsangi J.J., Wong S.Y. (2021). SARS-CoV-2 Vaccination in IBD: Past Lessons, Current Evidence, and Future Challenges. J. Crohn’s Colitis.

[6] Esposito S., Antoniol G., Labate M., Passadore L., Alvisi P., Dacco V., Ghizzi C., Colombo C., Principi N. (2021). Vaccines in Children with Inflammatory Bowel Disease: Brief Review. Vaccines.

[7] Turner D., Ruemmele F.M., Orlanski-Meyer E., Griffiths A.M., de Carpi J.M., Bronsky J., Veres G., Aloi M., Strisciuglio C., Braegger C.P. (2018). Management of Paediatric Ulcerative Colitis, Part 1: Ambulatory Care-An Evidence-based Guideline from European Crohn’s and Colitis Organization and European Society of Paediatric Gastroenterology, Hepatology and Nutrition. J. Pediatr. Gastroenterol. Nutr..

[8] Mack D.R., Benchimol E.I., Critch J., deBruyn J., Tse F., Moayyedi P., Church P., Deslandres C., El-Matary W., Huynh H. (2019). Canadian Association of Gastroenterology Clinical Practice Guideline for the Medical Management of Pediatric Luminal Crohn’ Disease. Gastroenterology.

[9] Harrington J.E., Hamilton R.E., Ganley-Leal L., Farraye F.A., Wasan S.K. (2020). The Immunogenicity of the Influenza, Pneumococcal, and Hepatitis B Vaccines in Patients with Inflammatory Bowel Disease Treated with Vedolizumab. Crohns Colitis 360.

[10] Kennedy N.A., Goodhand J.R., Bewshea C., Nice R., Chee D., Lin S., Chanchlani N., Butterworth J., Cooney R., Croft N.M. (2021). Anti-SARS-CoV-2 antibody responses are attenuated in patients with IBD treated with infliximab. Gut.

[11] Kennedy N.A., Lin S., Goodhand J.R., Chanchlani N., Hamilton B., Bewshea C., Nice R., Chee D., Cummings J.F., Fraser A. (2021). Infliximab is associated with attenuated immunogenicity to BNT162b2 and ChAdOx1 nCoV-19 SARS-CoV-2 vaccines in patients with IBD. Gut.

[12] Long M.D., Weaver K.N., Zhang X., Chun K., Kappelman M.D., Group P.-C.S. (2022). Strong Response to SARS-CoV-2 Vaccine Additional Doses Among Patients with Inflammatory Bowel Diseases. Clin. Gastroenterol. Hepatol..

[13] Shire Z., Reicherz F., Lawrence S.J., Sudan H., Golding L., Majdoubi A., Levett P., Lavoie P., Jacobson K. (2022). Antibody response to the BNT162b2 SARS-CoV-2 vaccine in paediatric patients with inflammatory bowel disease treated with anti-TNF therapy. Gut.

[14] Quan J., Ma C., Panaccione R., Hracs L., Sharifi N., Herauf M., Markovinovic A., Coward S., Windsor J.W., Caplan L. (2022). Serological responses to the first four doses of SARS-CoV-2 vaccine in patients with inflammatory bowel disease. Lancet Gastroenterol. Hepatol..

[15] Kennedy N.A., Janjua M., Chanchlani N., Lin S., Bewshea C., Nice R., McDonald T.J., Auckland C., Harries L.W., Davies M. (2023). Vaccine escape, increased breakthrough and reinfection in infliximab-treated patients with IBD during the Omicron wave of the SARS-CoV-2 pandemic. Gut.

[16] Jena A., James D., Singh A.K., Dutta U., Sebastian S., Sharma V. (2022). Effectiveness and Durability of COVID-19 Vaccination in 9447 Patients with IBD: A Systematic Review and Meta-Analysis. Clin. Gastroenterol. Hepatol..

[17] Ramos L., Hernandez-Porto M., Carrillo-Palau M., Alonso-Abreu I., Reygosa C., Hernandez-Guerra M. (2023). Impact of Biologic Agents on the Immune Response Induced by the Additional Dose of SARS-CoV-2 Vaccine in Inflammatory Bowel Disease Patients. Inflamm. Bowel Dis..

[18] Frey S., Chowdhury R., Connolly C.M., Werbel W.A., Segev D.L., Parian A.M., Ibd G. (2022). Antibody Response Six Months after SARS-CoV-2 mRNA Vaccination in Patients with Inflammatory Bowel Disease. Clin. Gastroenterol. Hepatol..

[19] Alexander J.L., Liu Z., Munoz Sandoval D., Reynolds C., Ibraheim H., Anandabaskaran S., Saifuddin A., Castro Seoane R., Anand N., Nice R. (2022). COVID-19 vaccine-induced antibody and T-cell responses in immunosuppressed patients with inflammatory bowel disease after the third vaccine dose (VIP): A multicentre, prospective, case-control study. Lancet Gastroenterol. Hepatol..

[20] Quan J., Ma C., Panaccione R., Hracs L., Sharifi N., Herauf M., Makovinovic A., Coward S., Windsor J., Caplan L. (2023). Serological responses to three doses of SARS-CoV-2 vaccination in inflammatory bowel disease. Gut.

[21] Bhurwal A., Mutneja H., Bansal V., Goel A., Arora S., Attar B., Minacapelli C.D., Kochhar G., Chen L.A., Brant S. (2022). Effectiveness and safety of SARS-CoV-2 vaccine in Inflammatory Bowel Disease patients: A systematic review, meta-analysis and meta-regression. Aliment. Pharmacol. Ther..

[22] Salinas G., De Rycke L., Barendregt B., Paramarta J., Hreggvidstdottir H., Cantaert T., van der Burg M., Tak P., Baeten D. (2013). Anti-TNF treatment blocks the induction of T cell-dependent humoral responses. Ann. Rheum. Dis..

[23] Edelman-Klapper H., Zittan E., Bar-Gil S., Rabinowitz K., Goren I., Avni-Biron I., Ollech J., Lichtenstein L., Banai-Eran H., Yanai H. (2022). Lower Serologic Response to COVID-19 mRNA Vaccine in Patients with Inflammatory Bowel Diseases Treated with Anti-TNFα. Gastroenterology.

[24] Lin S., Kennedy N.A., Saifuddin A., Sandoval D.M., Reynolds C.J., Seoane R.C., Kottoor S.H., Pieper F.P., Lin K.M., Butler D.K. (2022). Antibody decay, T cell immunity and breakthrough infections following two SARS-CoV-2 vaccine doses in inflammatory bowel disease patients treated with infliximab and vedolizumab. Nat. Commun..

[25] Liu Z., Le K., Zhou X., Alexander J.L., Lin S., Bewshea C., Chanchlani N., Nice R., McDonald T.J., Lamb C.A. (2023). Neutralising antibody potency against SARS-CoV-2 wild-type and omicron BA.1 and BA.4/5 variants in patients with inflammatory bowel disease treated with infliximab and vedolizumab after three doses of COVID-19 vaccine (CLARITY IBD): An analysis of a prospective multicentre cohort study. Lancet Gastroenterol. Hepatol..

[26] Lee K.J., Choi S.Y., Lee Y.M., Kim H.W. (2022). Neutralizing Antibody Response, Safety, and Efficacy of mRNA COVID-19 Vaccines in Pediatric Patients with Inflammatory Bowel Disease: A Prospective Multicenter Case-Control Study. Vaccines.

[27] Alexander J.L., Kennedy N.A., Ibraheim H., Anandabaskaran S., Saifuddin A., Castro Seoane R., Liu Z., Nice R., Bewshea C., D’Mello A. (2022). COVID-19 vaccine-induced antibody responses in immunosuppressed patients with inflammatory bowel disease (VIP): A multicentre, prospective, case-control study. Lancet Gastroenterol. Hepatol..

[28] Hall V., Ferreira V., Ku T., Ierullo M., Majchrzak-Kita B., Chaparro C., Selzner N., Schiff J., McDonald M., Tomlinson G. (2021). Randomized trial of a third dose of mRNA-1273 vaccine in transplant recipients. N. Engl. J. Med..

[29] Lin S., Lau L.H., Chanchlani N., Kennedy N.A., Ng S.C. (2022). Recent advances in clinical practice: Management of inflammatory bowel disease during the COVID-19 pandemic. Gut.

[30] Dailey J., Kozhaya L., Dogan M., Hopkins D., Lapin B., Herbst K., Brimacombe M., Grandonico K., Karabacak F., Schreiber J. (2022). Antibody Responses to SARS-CoV-2 After Infection or Vaccination in Children and Young Adults with Inflammatory Bowel Disease. Inflamm. Bowel Dis..

[31] Shiga H., Kakuta Y., An K., Abe Y., Fujimaki S., Shimoyama Y., Naito T., Moroi R., Kuroha M., Khor S.S. (2023). Response to COVID-19 vaccine is reduced in patients with inflammatory bowel disease, but improved with additional dose. J. Gastroenterol. Hepatol..

[32] Kasti A., Weaver K.N., Zhang X., Strople J.A., Adler J., Dubinsky M., Bousvaros A., Watkins R., Dai X., Chen W. (2023). Humoral immune response and safety of SARS-CoV-2 vaccination in pediatric inflammatory bowel disease. Am. J. Gastroenterol..

[33] Ungaro R.C., Brenner E.J., Agrawal M., Zhang X., Kappelman M.D., Colombel J.F., Surveillance Epidemiology of Coronavirus Under Research Exclusion for Inflammatory Bowel Disease (SECURE-IBD) Research Group (2022). Impact of Medications on COVID-19 Outcomes in Inflammatory Bowel Disease: Analysis of more than 6000 Patients From an International Registry. Gastroenterology.

[34] Brenner E.J., Pigneur B., Focht G., Zhang X., Ungaro R.C., Colombel J.F., Turner D., Kappelman M.D., Ruemmele F.M. (2021). Benign Evolution of SARS-CoV2 Infections in Children with Inflammatory Bowel Disease: Results from Two International Databases. Clin. Gastroenterol. Hepatol..

[35] Brenner E.J., Ungaro R.C., Gearry R.B., Kaplan G.G., Kissous-Hunt M., Lewis J.D., Ng S.C., Rahier J.F., Reinisch W., Ruemmele F.M. (2020). Corticosteroids, But Not TNF Antagonists, Are Associated with Adverse COVID-19 Outcomes in Patients With Inflammatory Bowel Diseases: Results from an International Registry. Gastroenterology.

[36] Hadi Y.B., Thakkar S., Shah-Khan S.M., Hutson W., Sarwari A., Singh S. (2021). COVID-19 Vaccination Is Safe and Effective in Patients with Inflammatory Bowel Disease: Analysis of a Large Multi-institutional Research Network in the United States. Gastroenterology.

[37] Lev-Tzion R., Focht G., Lujan R., Mendelovici A., Friss C., Greenfeld S., Kariv R., Ben-Tov A., Matz E., Nevo D. (2022). COVID-19 Vaccine Is Effective in Inflammatory Bowel Disease Patients and Is Not Associated with Disease Exacerbation. Clin. Gastroenterol. Hepatol..

[38] Mujukian A., Kumar R., Li D., Debbas P., Botwin G.J., Cheng S., Ebinger J., Braun J., McGovern D., Melmed G.Y. (2023). Postvaccination Symptoms After SARS-CoV-2 mRNA Vaccination Among Patients with Inflammatory Bowel Disease: A Prospective, Comparative Study. Inflamm. Bowel Dis..

[39] Weaver K.N., Zhang X., Dai X., Watkins R., Adler J., Dubinsky M.C., Kastl A., Bousvaros A., Strople J.A., Cross R.K. (2022). Impact of SARS-CoV-2 Vaccination on Inflammatory Bowel Disease Activity and Development of Vaccine-Related Adverse Events: Results From PREVENT-COVID. Inflamm. Bowel Dis..

[40] Shrestha L.B., Foster C., Rawlinson W., Tedla N., Bull R.A. (2022). Evolution of the SARS-CoV-2 omicron variants BA.1 to BA.5: Implications for immune escape and transmission. Rev. Med. Virol..

[41] Woelfel S., Dutschler J., Konig M., Dulovic A., Graf N., Junker D., Oikonomou V., Krieger C., Truniger S., Franke A. (2023). STAR SIGN study: Evaluation of COVID-19 vaccine efficacy against the SARS-CoV-2 variants BQ.1.1 and XBB.1.5 in patients with inflammatory bowel disease. Aliment. Pharmacol. Ther..

[42] Bellusci L., Zahra F.T., Hopkins D.E., Salazar J.C., Hyams J.S., Khurana S. (2022). Durability of Immunity Is Low Against Severe Acute Respiratory Syndrome Coronavirus 2 Omicron BA.1, BA.2, and BA.3 Variants After Second and Third Vaccinations in Children and Young Adults with Inflammatory Bowel Disease Receiving Biologics. Gastroenterology.

[43] WHO (2023). WHO Roadmap on Uses of COVID-19 Vaccines in the Context of Omicron and High Population Immunity.

