# Supplementary material for: Durability of Antibody Responses to SARS-CoV-2 Vaccination over 12 Months in Pediatric Inflammatory Bowel Disease
Source: Vaccines (Basel). 2025 May 22;13(6):549. doi: 10.3390/vaccines13060549 (PMC12197349; doi:10.3390/vaccines13060549)
Supplement: Supplementary file 1 [file vaccines-13-00549-s001.zip › vaccines-3621112-supplementary.pdf]

vaccines

SUPPLEMENTARY MATERIAL

Table S1. Anti-SARS-CoV-2 spike and RBD Ancestral IgG levels 3 and 12 months after the 1st dose of SARS-CoV-2 vaccine by age groups. Descriptive analysis of geometric mean and geometric standard deviation of antibody levels in AU/mL from MSD spike assay. Timepoints correspond to 3 months (mean 90 days) and 12 months (mean 360 days) after receiving the first dose of SARS-CoV2 vaccine. N=X-X indicates total samples available for each timepoint (3 months and 12 months respectively). The Meso Scale Discovery assay threshold for antibody positivity is defined by the manufacturer as 1,960AU/mL for spike and 538 AU/mL for RBD.

		Children <12 years (N=29,25)	Adolescents 12-18 years (N=116,119)
**Anti-spike IgG levels (AU/mL)**	**3 months**	77,220 (5.8)	115,994 (4.3)
	**Mean ratio (95% CI)**	0.66 (0.33-1.36)	Reference
	**12 months**	47,436 (5.5)	172,514 (5.1)
	**Mean ratio (95% CI)**	**0.27 (0.13-0.58)***	Reference
**Anti-RBD S1 IgG levels (AU/mL)**	**3 months**	35,408 (6.5)	60,923 (3.4)
	**Mean ratio (95% CI)**	0.32 (0.05-2.11)	Reference
	**12 months**	21,512 (3.7)	83,057 (3.9)
	**Mean ratio (95% CI)**	0.59 (0.21-1.70)	Reference
**Anti-spike BA.5 IgG levels (AU/mL)$**	**3 months**	Not available	53,626 (4.0)
	**Mean ratio (95% CI)**	-	Reference
	**12 months**	15,726 (5.7)	32,477 (5.9)
	**Mean ratio (95% CI)**	0.48 (0.16 – 1.49)	Reference

* p<0.05; **p<0.001 (compared to vedolizumab group) calculated on log-transformed data with 2-sided unpaired t-test with Bonferroni's correction for multiple comparisons. Mean ratio represents the geometric mean ratio, the relative difference between groups, obtained from the difference of means in the log-transformed data. $ based on available samples for each age group (<12 years: N=0,22; 12-18: N=61,49).

Table S2. Seroconversion rates for Anti-SARS-CoV-2 spike and RBD IgG Ancestral levels at 3 and 12 months after 1st Dose of SARS-CoV-2 vaccine. Cut-off values established by MesoScale Discovery assay to detect positive samples (1,960 AU/mL for Spike and 538 AU/mL for RBD). N=X-X indicates total samples available for each timepoint (3 months and 12 months respectively). Anti-TNF-IM" for anti-TNF (infliximab or adalimumab) combined with immunomodulator (azathioprine or methotrexate); "Anti-TNF" for Anti-TNF (infliximab or adalimumab) in monotherapy; "vedolizumab" for vedolizumab in mono or combination therapy and "steroids" for prednisone at any dose in monotherapy or in combination with any other medication.

	Anti-TNF-IM (N=63-64)	Anti-TNF (N=60-57)	Vedolizumab (N=14-11)	Steroids (N=8-12)
**Spike 3 months**	63 (100%)	59 (98.3%)	7 (87.5%)	14 (100%)
**Spike 12 months**	63 (98.4%)	55 (96.5%)	11 (91.7%)	11 (100%)
**RBD 3 months**	63 (100%)	60 (98.4%)	7 (87.5%)	14 (100%)
**RBD 12 months**	64 (98.5%)	55 (96.5%)	12 (92.3%)	11 (100%)
